# We-VoltamoStat: A wearable potentiostat for voltammetry analysis with a smartphone interface

**DOI:** 10.1016/j.ohx.2023.e00441

**Published:** 2023-06-13

**Authors:** Nur Fatin Adini Ibrahim, Anas Mohd Noor, Norhayati Sabani, Zulkarnay Zakaria, Asnida Abdul Wahab, Asrulnizam Abd Manaf, Shazlina Johari

**Affiliations:** a Faculty of Electronic Engineering & Technology (FKTEN), Universiti Malaysia Perlis, Arau 02600, Malaysia; b Center of Excellance Micro System Technology (MicTEC), Universiti Malaysia Perlis, Arau 02600, Malaysia; c Sports Engineering Research Center (SERC), Universiti Malaysia Perlis, Arau 02600, Malaysia; d Department of Biomedical Engineering and Health Sciences, Universiti Teknologi Malaysia, Johor, Bahru 81310, Malaysia; e Collaborative Microelectronic Design Excellence Centre (CEDEC), Universiti Sains Malaysia, Bayan Lepas 11900, Malaysia

**Keywords:** Potentiostat, Wearable device, Voltammetry, Wearable electrochemical sensing

## Abstract

Wearable technology, such as electronic components integrated into clothing or worn as accessories, is becoming increasingly prevalent in fields like healthcare and biomedical monitoring. These devices allow for continuous monitoring of important biomarkers for medical diagnosis, monitoring of physiological health, and evaluation. However, an open-source wearable potentiostat is a relatively new technology that still faces several design limitations such as short battery lifetime, bulky size, heavy weight, and the requirement for a wire for data transmission, which affects comfortability during long periods of measurement. In this work, an open-source wearable potentiostat device named We-VoltamoStat is developed to allow interested parties to use and modify the device for creating new products, research, and teaching purposes. The proposed device includes improved and added features, such as wireless real-time signal monitoring and data collection. It also has an ultra-low power consumption battery estimated to deliver 15 mA during operating mode for 33 h and 20 min and 5 mA during standby mode for 100 h without recharging. Its convenience for wearable applications, tough design, and compact size of 67x54x38 mm make it suitable for wearable applications. Cost-effectiveness is another advantage, with a price less than 120 USD. Validation performance tests indicate that the device has good accuracy, with an R2 value of 0.99 for linear regression of test accuracy on milli-, micro-, and nano-Ampere detection. In the future, it is recommended to improve the design and add more features to the device, including new applications for wearable potentiostats.

## Graphical abstract

**Figure:**
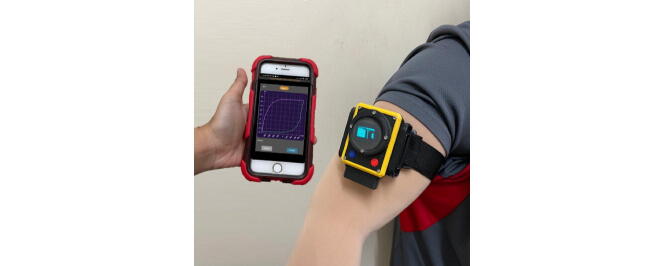

**Specifications table**

**Table:** 

Hardware name	We-VoltamoStat
Subject area	Chemistry and Biochemistry
Hardware type	• Measuring physical properties and in-lab sensors • Electrical engineering and computer science
Open-Source License	CC BY 4.0
Cost of Hardware	114.44 USD
Source File Repository	https://doi.org/10.17632/266vrryspz.3

## Hardware in context

The growth of open-source potentiostats development has been significant, with many free hardware designs and source codes readily available to the public. This enables greater collaboration and innovation in the development of the device, as well as increased transparency and security. Users can use, modify, and distribute the device. These potentiostats offer lower cost builds and improved design components that are miniature and lightweight compared to commercially available potentiostats, which makes them useful for research, product development, and educational purposes, such as TBISTAT [1], PassStat [2], and SweepStat [3]. However, most of the open-source potentiostats such as KickStat [4], EduPotStat [5], MYSTAT [6] and others previously reported [7], [8], [9], [10] require a wired connection which is not practical for wearable applications. For example, real-time human biofluid biochemical analysis requires a portable and wearable system. A smartphone can be a convenient alternative to a computer or laptop as a user interface because it is portable and easily fits in a pocket. Additionally, monitoring the outputs can be done through an app which is more convenient.

Wireless communications between smartphones and potentiostats, such as Bluetooth [11], [12], [13], [14], [15], [16], [17] and Wi-Fi [18], [19], [20], [21], are widely used for data processing, user interfacing, data collection, and data sharing. Many studies choose Bluetooth as their communication method because it allows for wireless data transmission, has lower power consumption, supports standard protocols, is easy to use, does not require an internet connection, and provides stable and strong signals over long distances. Recent studies on open-source potentiostats often introduce their specific functions, but none of them have additional I/O ports that allow connection to other external devices, which would enable multiple tasks at the same time [22], [23], [24]. For example, Azimi et al. developed a system for a biosensing application using a potentiostat and syringe pump connected to a microfluidic device for rapid and accurate glucose detection platforms [25]. However, the elements of the system are powered by different power sources and controllers, leading to the need for multiple connections and reducing the device's design for simplification, compactness, and portability. In addition, some studies have proposed additional controlling ports on their devices for controlling a micropump, but these devices are bulky, require wires for data transfer and power-up, only serve a specific mode of operation, and are not suitable for wearable applications [26], [27], [28].

This work presents the development of an open-source wearable potentiostat for electrochemical analysis applications with a smartphone interface. The device addresses design deficiencies in existing reported open-source potentiostats and offers low-cost development, easy maintenance, and the ability to be upgraded. The separated electronic circuit boards system allows for easy upgrading with new functionality and reprogramming with linear sweep (LSV) and cyclic voltammetry (CV) measurement modes. The We-Voltamostat design is durable for rough environments and has user-friendly features that make it suitable for wearable applications, such as sweat analysis. The device demonstrates high accuracy and can detect down to nano-ampere levels for reliable measurements and wide application use.

## Hardware description

**Fig. 1:**
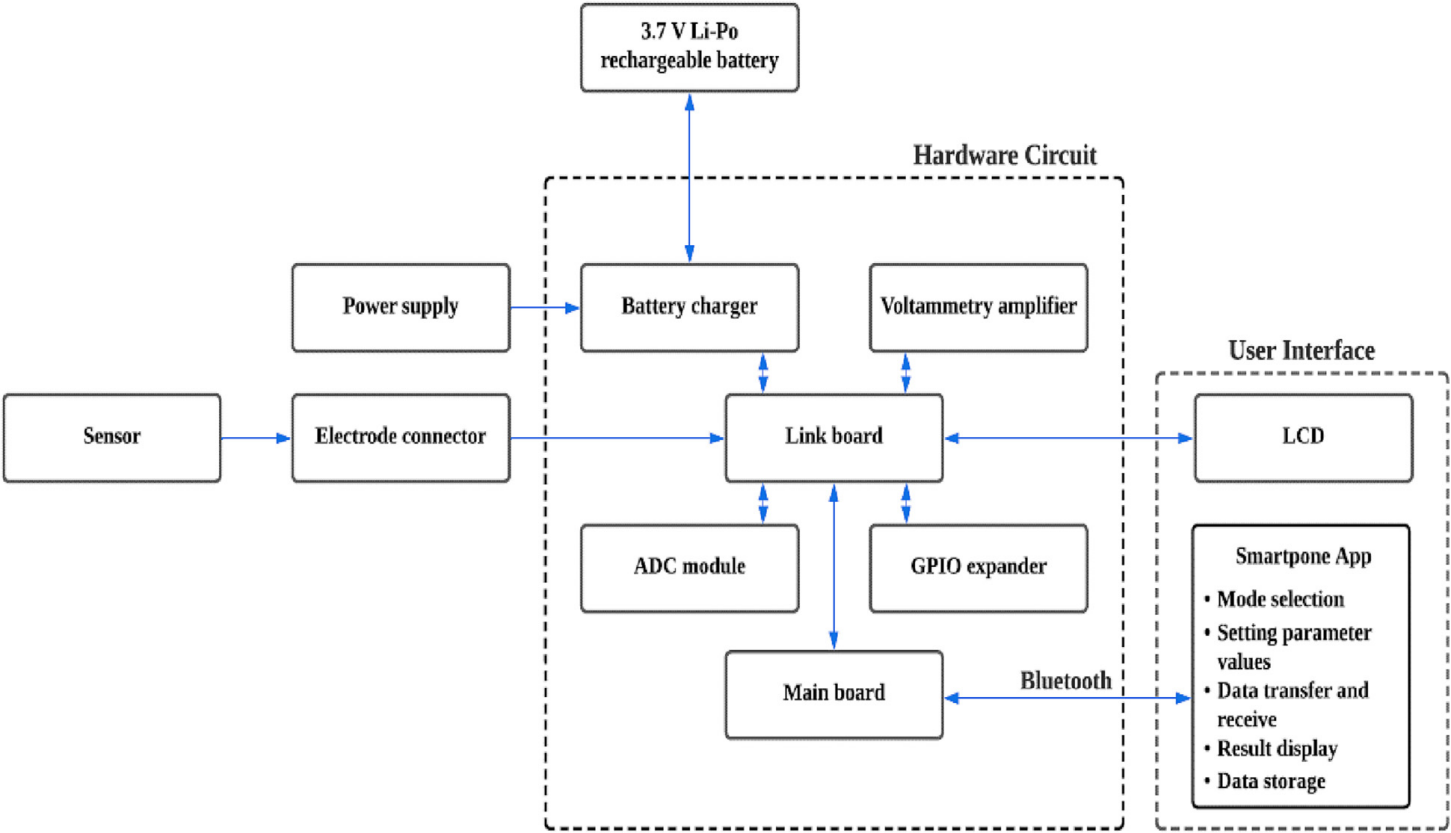
Functional block diagram of We-VoltamoStat.

In this section, the schematic diagrams of the sub-circuits of the We-VoltamoStat's printed circuit boards (PCBs) are thoroughly explained. The device consists of six PCBs, each performing a specific function, such as a battery charger circuit, a general-purpose input/output (GPIO) expander, an analogue-to-digital converter (ADC) module, a main board, a voltammetry amplifier circuit, and a link board. The boards are designed with pins and connectors that allow for simple integration and easy maintenance for future upgrades. The functional block diagram of the We-VoltamoStat's is shown in Fig. 1.

**Fig. 2:**
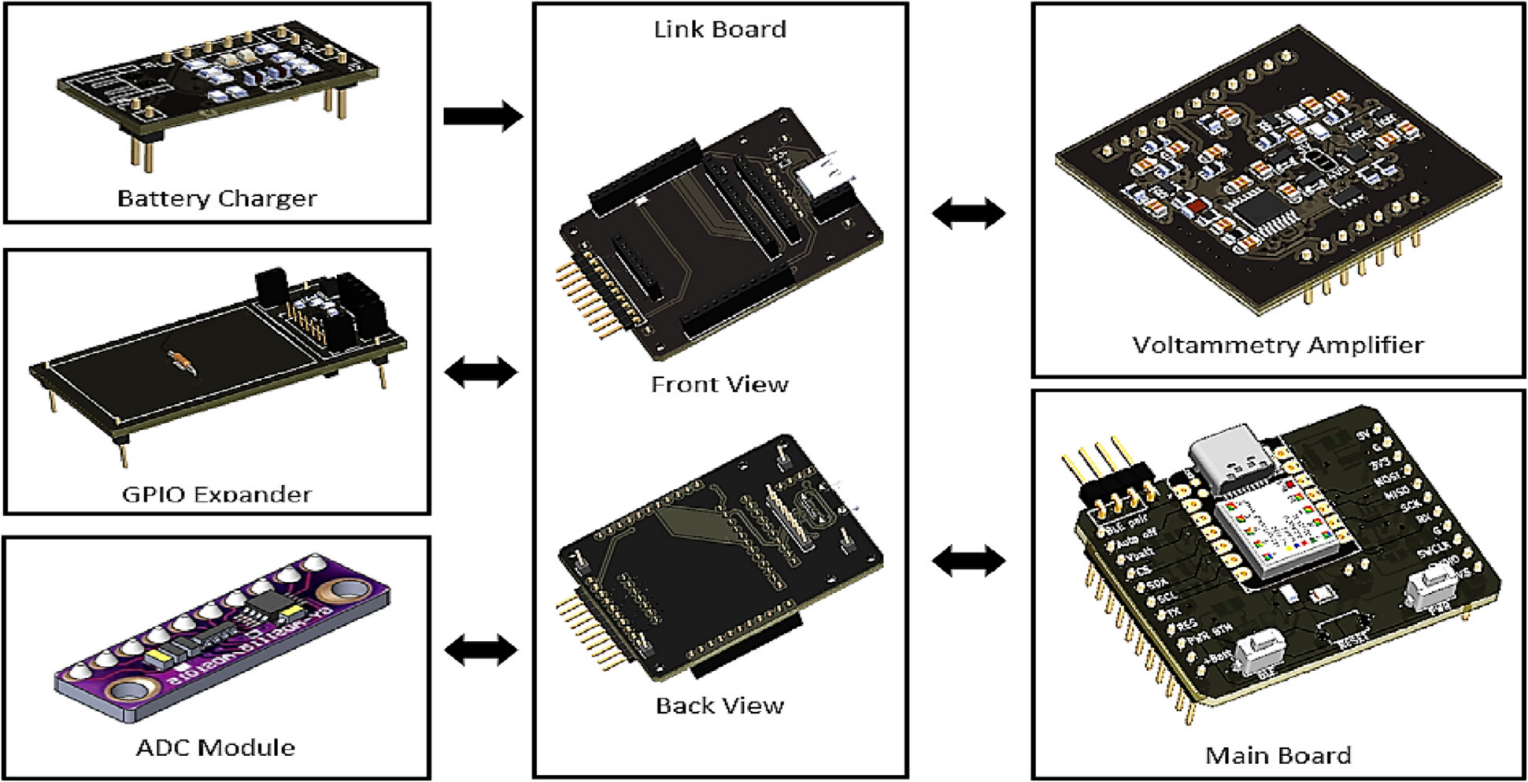
We-VoltamoStat printed circuit boards.

The following subtopics explain the schematic diagrams and circuit design considerations for each block diagram depicted in Fig. 1. Additionally, Fig. 2 provides images of the We-VoltamoStat PCBs.

• Battery Charger Circuit Board

**Fig. 3:**
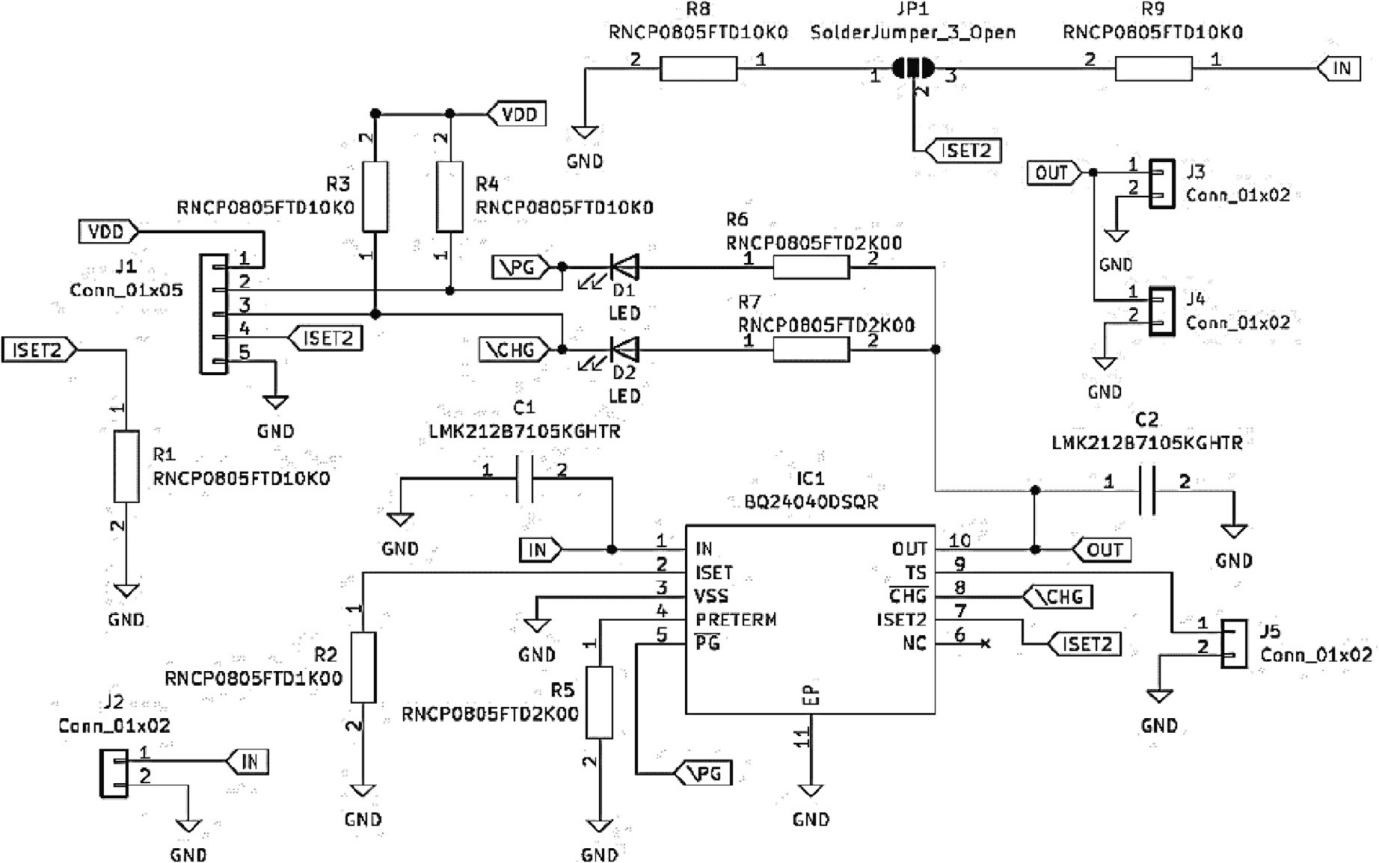
Schematic diagram for battery charger.

A rechargeable battery is preferred for powering electronic devices due to its portability, reusability, and ability to be recharged, making it ideal for wearable applications. The DSQR IC chip (from Texas Instruments) is used to implement charge control and management functions in the We-VoltamoStat. This chip was selected for its high efficiency, which minimizes power loss and prolongs battery life, as well as its compact size, safety features, and flexibility with different types of rechargeable batteries. The battery charger circuit was developed based on the manufacturer's recommendations, as shown in Fig. 3. Additionally, capacitors (C1 and C2) are placed between the input and ground to form a ground connection in a capacitor, which removes electrical noise while maintaining a stable signal. When a high input voltage source is detected, the power good indication (PG) terminal will turn on and provide a low-resistance path back to the ground, reducing the overvoltage. The R2 resistor controls the output current in ISET, and it functions as a short-protected, which means that if a short occurs, at least 80 mA of output current, the IC charge will turn off and can only be reset by charging a low power. The charge (CHG) terminal will also reset and begin charging the battery when the thermistor (TS) terminal is pulled down and the pre-charge mode is reduced. When the current flowing through the battery reaches 10% of the programmed charge current, this charge terminal will automatically shut-off. Meanwhile, the TS drives the temperature conditions to follow the new JEITA temperature standard for Li-Ion and Li-Pol batteries. The charge current of the BQ24040DSQR IC chip is cutoff if the internal temperature of the IC junction threshold is exceeded by 150 °C.

• GPIO expander circuit board

**Fig. 4:**
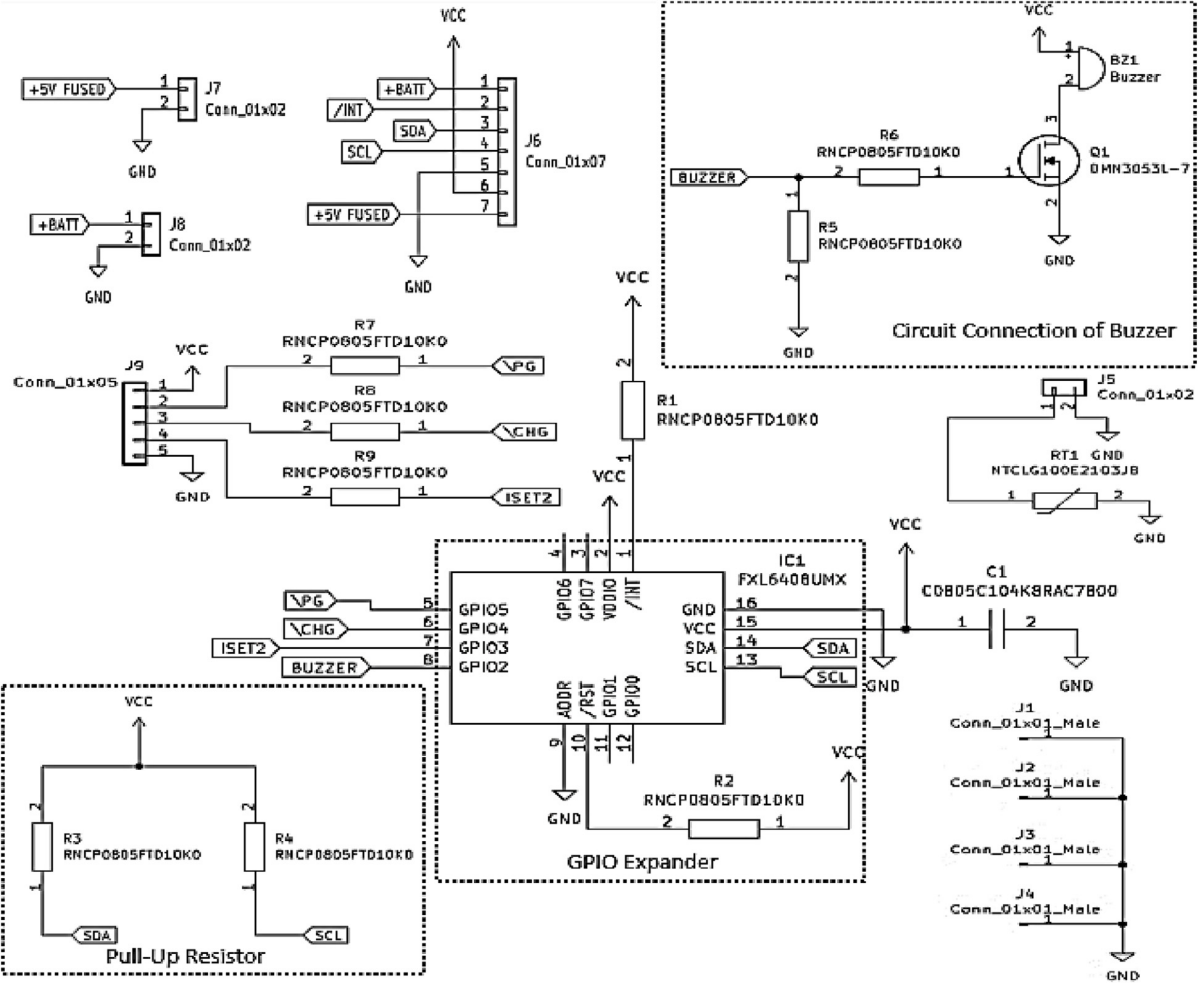
Schematic diagram for GPIO expander.

The GPIO expander offers a simple, cost-effective, and broad selection of options to add extra inputs and outputs (I/O) for controlling multiple peripheral signals. The FXL6408UMX IC chip (Onsemi, USA) is used to specify the functions of the 4-pin I/O, such as the buzzer, PG CHG, and ISET2, to monitor the status of a battery charger. This IC chip is equipped with an 8-bit GPIO expander that is controlled by I2C. Pull-up resistors (R3 and R4) are added to maintain a good signal. Fig. 4 shows the schematic diagram of the GPIO expander, which includes a circuit for the GPIO expander and a buzzer circuit. The FXL6408 controls these input mode GPIO pins of PG, CHG, and ISET2 through the interrupt (/INT) pin. The buzzer operation will be controlled by the microcontroller to produce a beep sound, notifying the presence of voltages passing through it when the system is powered-up. In addition, a thermistor, RT1, is installed on the battery attachment to monitor the battery's temperature.

• ADC module circuit board

**Fig. 5:**
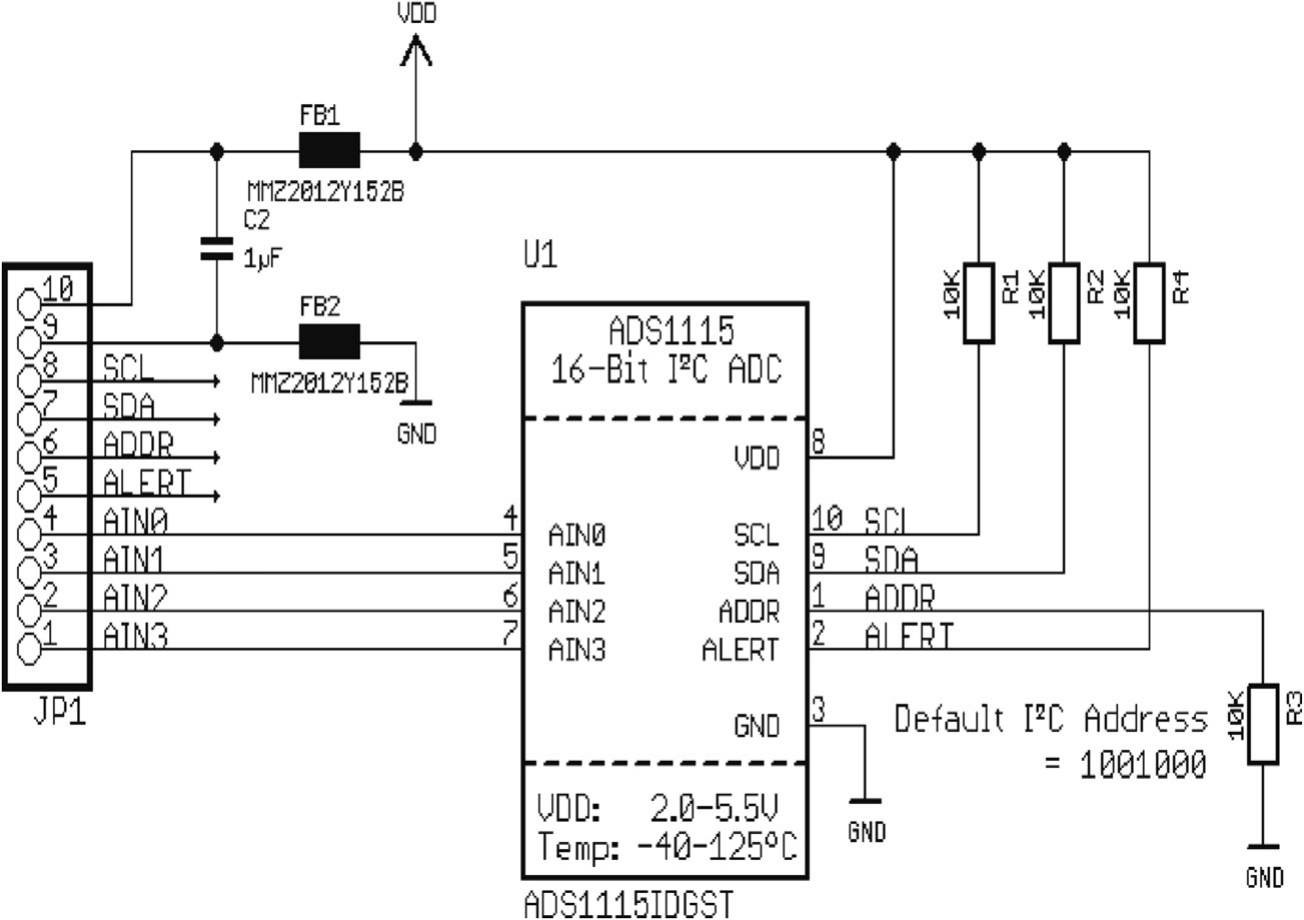
Schematic diagram of ADC module.

The ADS1115 module is utilized to provide high-resolution ADC, which can overcome the limitations of the built-in ADC in microcontrollers. General microcontrollers have a resolution of no more than 12-bits, which is not sufficient, especially for measuring nano-level signals. This module is used for internal WE-VoltamoStat calibration and measuring voltammetry current and voltage. Fig. 5 shows the schematic diagram of the commercial ADS1115 module, which consists of an ADS1115 IC chip (from Texas Instruments), ferrites chips (FB1 and FB2), pull-up resistors (R1, R2, and R4), and a pull-down resistor (R3). The ADS1115 IC chip is selected for its high resolution of 16-bits, low-power consumption, and a programmable gain amplifier for a wide range of measurements. It has four analog input channels that can be configured as follows: AIN0 to “virtual ground,” AIN1 to “AMP V OUT,” AIN2 to “V SOURCE,” and AIN3 to “MILLI AMPERE V OUT.” These inputs are then capable of accurately converting analog to digital signals over a broader voltage or current range. These input pins, together with VDD, GND, SCL, SDA, ADDR, and ALERT of the ADC module, are integrated into the link board. The FB1 and FB2 are added to the ADC module board to smooth out the voltages.

• Voltammetry amplifier circuit board

**Fig. 6:**
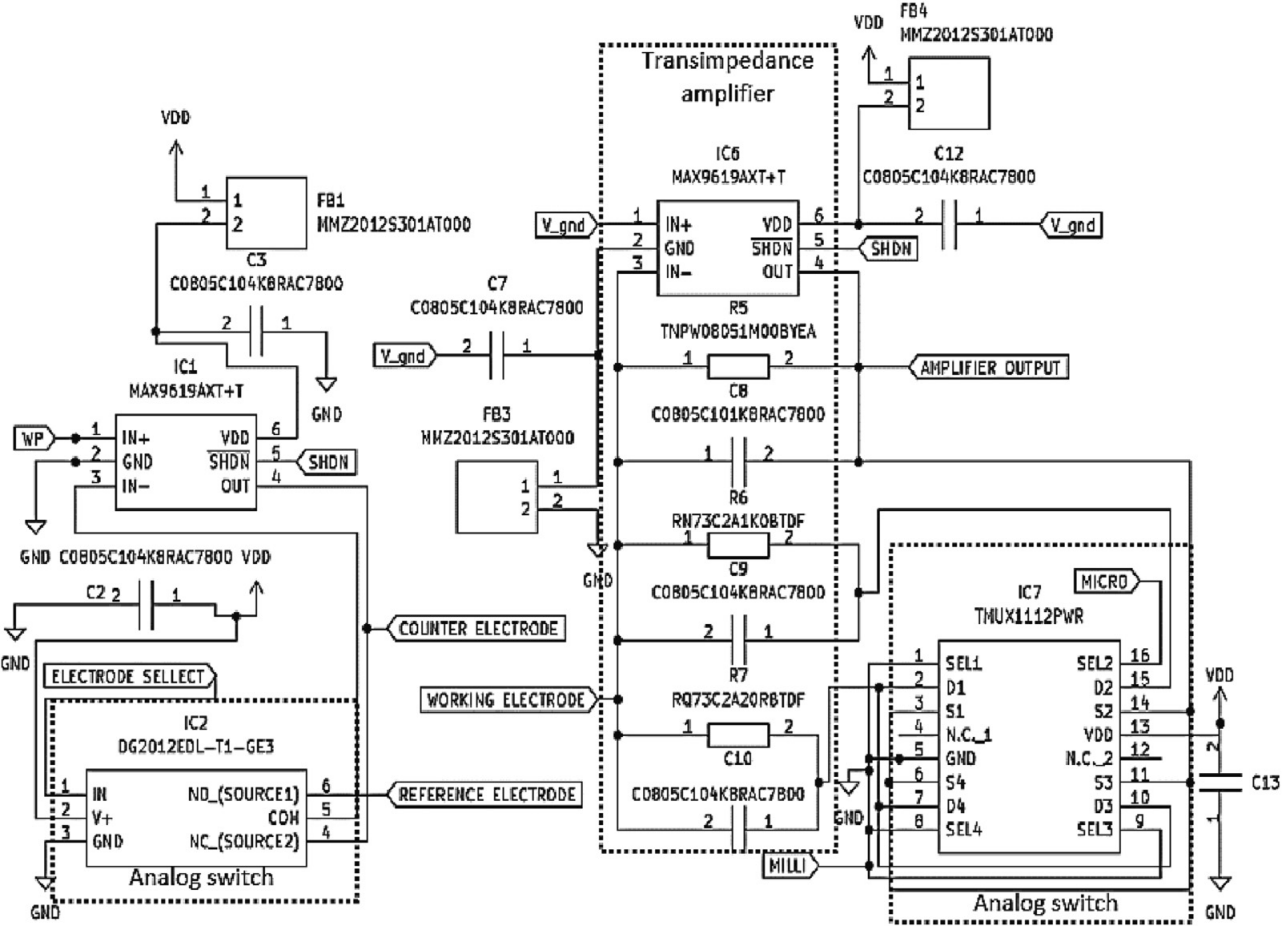
Schematic diagram of voltammetry amplifier circuit board.

A voltammetry amplifier is a device used to amplify the small current signals produced by a sensor. It is use on a working electrode (WE), a reference electrode (RE), and a counter electrode (CE) circuits. Even though there are a few available commercial dedicated ICs (e.g., LMP91000 and ADuCM355), these components functionality cannot be upgraded such as adding more sensing channels, functions, and voltammetry measurement modes, which could limit the device's applications. A circuit connection for each electrode is shown in Fig. 6. The circuit includes analogue switch, precision operational amplifier (op-amp) and precision switch multiplexer.

The DG2012E (Vishay Siliconix, USA) is used because of its fast-switching speed and a high-performance single-pole double-throw (SPDT) analogue switch. This SPDT switch can connect to any two paths circuit selected, which pin NO_source1 connect with RE, and NC_source2 connect with CE. The RE is used to measure the WE potential. Meanwhile, the CE is a conductor that completes the cell circuit to measure the WE current. When the DG2012E IC chip is connected to the RE, it is nearly infinite input resistance resulting no significant current transfer at this electrode. Its circuit keeps the RE's potential stable for measurement potential difference between the WE and RE. When a signal is applied at the wiper (WP) to an electrochemical cell at the CE with respect to the RE, it supplies a current infinitely. It then provides a current path that shall be measured between the CE and WE. The WP signal input is also used to control the potential difference between the WE and RE. The potential difference between the WE and the RE is continuously managed because the WE is kept at stable potential (pseudo ground) by managing the polarization of the CE.

The MAX9619 (Analog Device, USA) is chosen because of a high-performance, low-power, and low-noise op-amp design for signal processing and sensor interface. The op amp has a high input impedance of 1.1MΩ and a low output impedance of 2.5 Ω, making it ideal for use in applications that require high accuracy and low noise. The MAX9619 can be used to configure as a transimpedance amplifier (TIA) by connecting a feedback resistor for generating an output voltage proportionate to an input current and a feedback capacitor for maintaining the signal's stability. The TIA gain is set by the value of the feedback resistors. The design circuit of TIA in this work is able to measure from nano-amperes to micro-amperes by selecting the feedback resistors, where R5 measures the current in the nano-ampere range, R6 measures the current in the micro-ampere, and R7 measures the current in the milli-ampere range. Thus, the presetting resistors in the design gives users advantages in selecting a suitable sensing range with high-accuracy detection. Additionally, by using a low-noise op amp such as the MAX9619, the TIA circuit can reduce the amount of noise present in the measurements, resulting in even more accurate, stable signal and precise results.

The TMUX1112 (Texas Instrument, USA) is preferred for signal selections and switching with low-power consumption of four independently selectable single-poles. It features a low on-resistance of 30 O and a low leakage current of 1 pA. It also has a high input impedance of $10^{12}$ ohms, making it suitable for use in applications such as data acquisition and signal routing. The current range selection by TIA is performed by the analogue switch of the TMUX1112PWR IC chip. Furthermore, to improve the noise immunity in the circuit, MMZ2012 ferrite beads are used as a passive filter to suppress noise and block unwanted electromagnetic interference (EMI) and radio frequency interference (RFI) from entering or leaving the circuit.

**Fig. 7:**
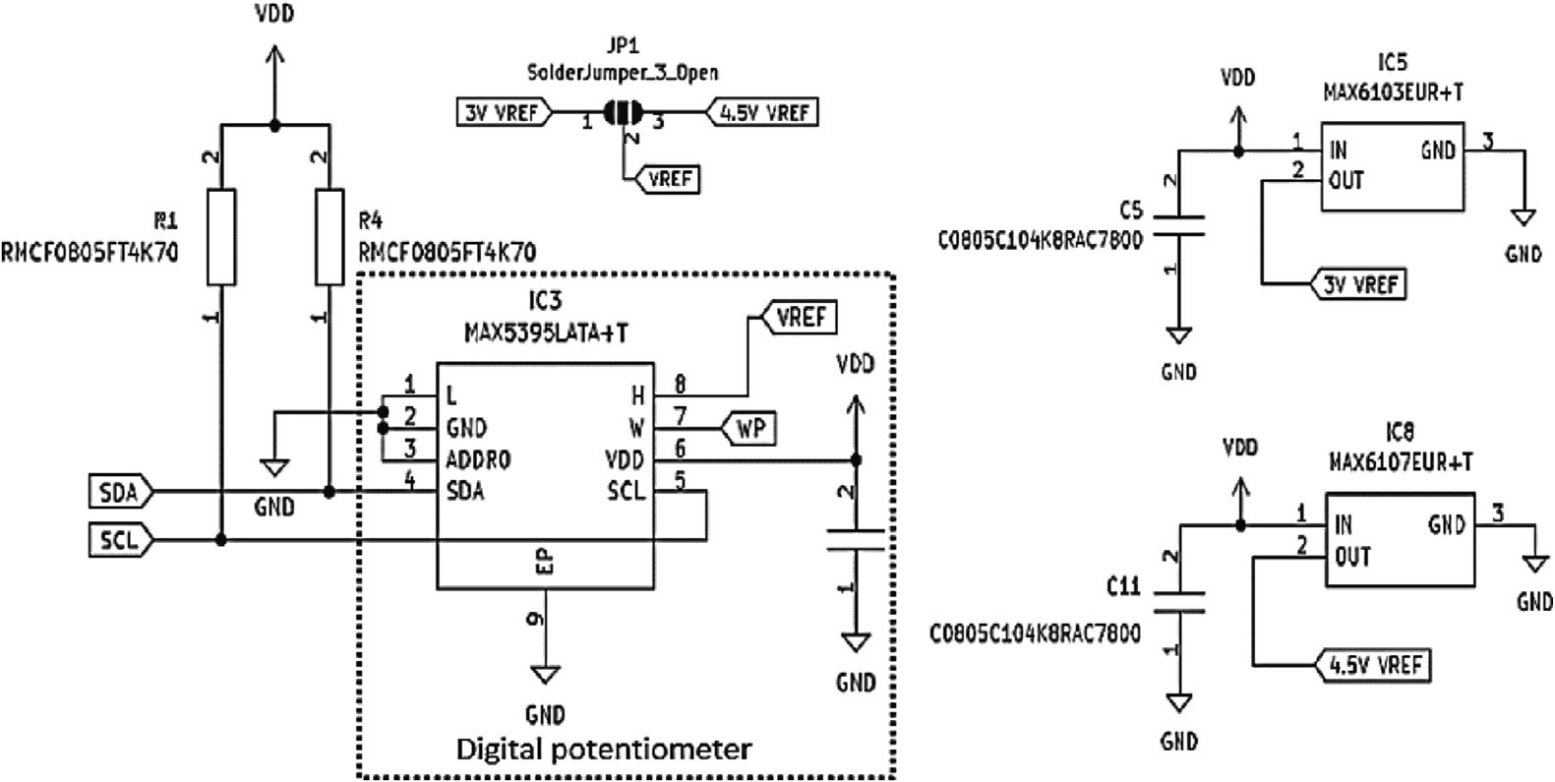
Terminal reference circuit diagram of VREF, 3VREF and 4.5VREF.

**Fig. 8:**
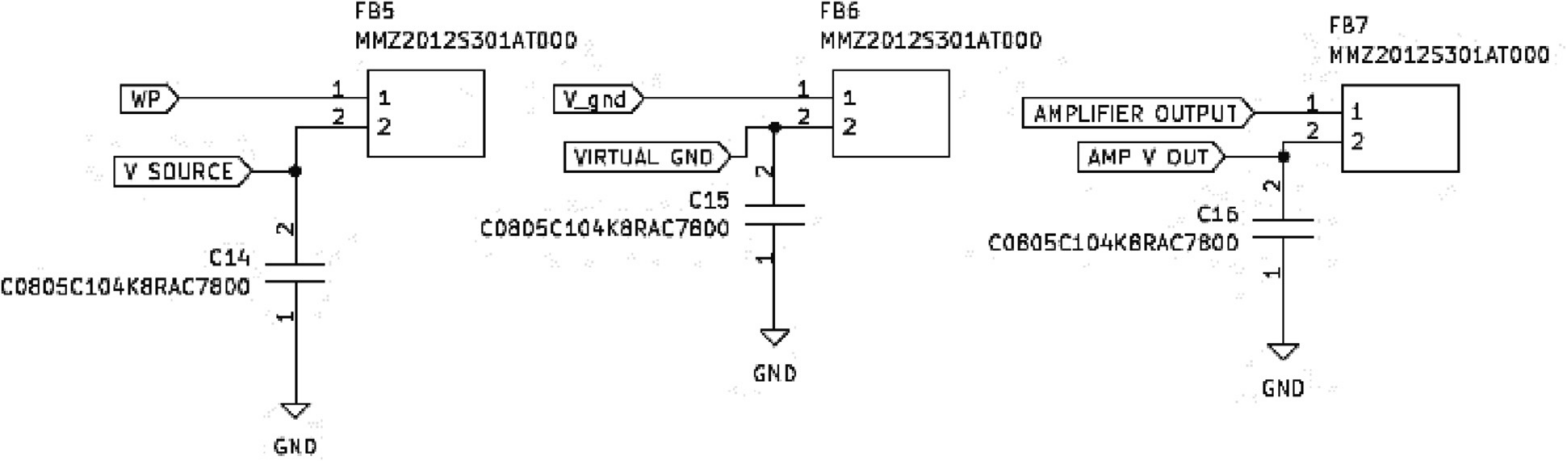
Schematic diagram of noise removal.

In the voltammetry amplifier, there is also a terminal reference of VREF, 3VREF, and 4.5VREF, as shown in Fig. 7. The MAX5395 digital potentiometer voltage-controlled resistor is selected to provide multiple and variable reference voltage sources with a higher resolution of 256 steps (8 bits). It also could be controlled using $I^{2}$ C. Additional series resistors are added in line with SDA and SCL to ensure that the line maintains a high logic level when no other device is actively driving the line. Without the pull-up resistor, the line could float to an indeterminate state and cause communication errors. An additional capacitance load has been added at the supply voltage line for filter purpose. Fig. 8 shows an additional ferrite beads circuit using MMZ2012 (TDK Corporation, Japan), and load capacitors as a filter are added to each input, output, and bias voltage of the voltammetry amplifier circuit.

• Main board

**Fig. 9:**
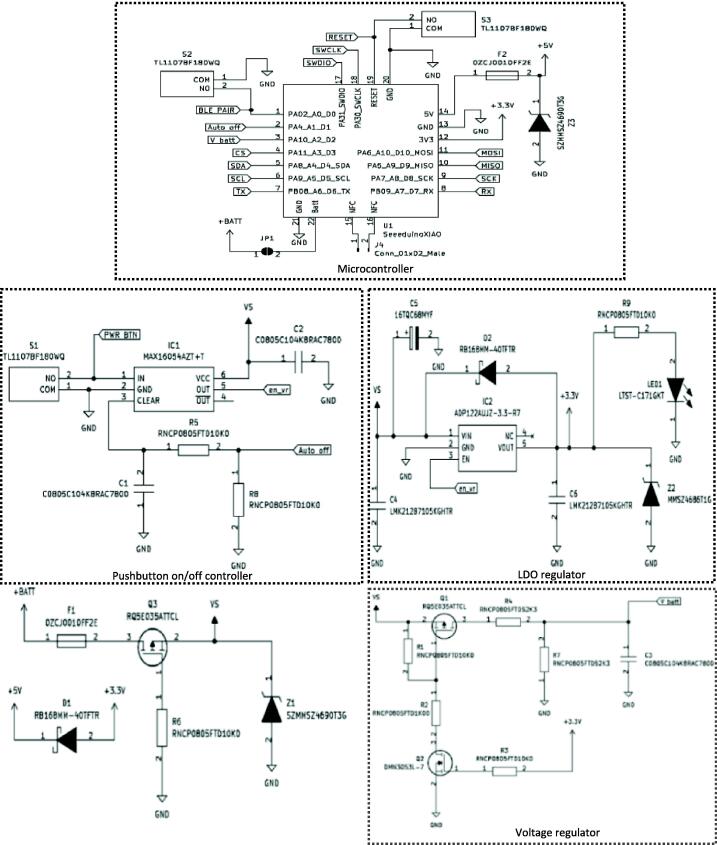
Schematic diagram of main board.

The main board circuit consists of a microcontroller (Seeeduino Xiao), voltage regulator circuitry and switches. The Seeeduino XIAO controls the entire system function and performs power management. This microcontroller is chosen over other available microcontrollers because it is a compact, low-cost, low-power consumption, and already have Bluetooth module onboard that is suitable for wearable applications. The voltage regulators and low drop-out (LDO) regulators are used to reduce the input voltage and maintain a constant output voltage to monitor power loss. Three switches (TL1107 type) are added to the main board: S1 for the push button on/off controller, S2 for the push button on/off Bluetooth, and S3 for the push button on/off reset, as shown in Fig. 9.

• Link board

**Fig. 10:**
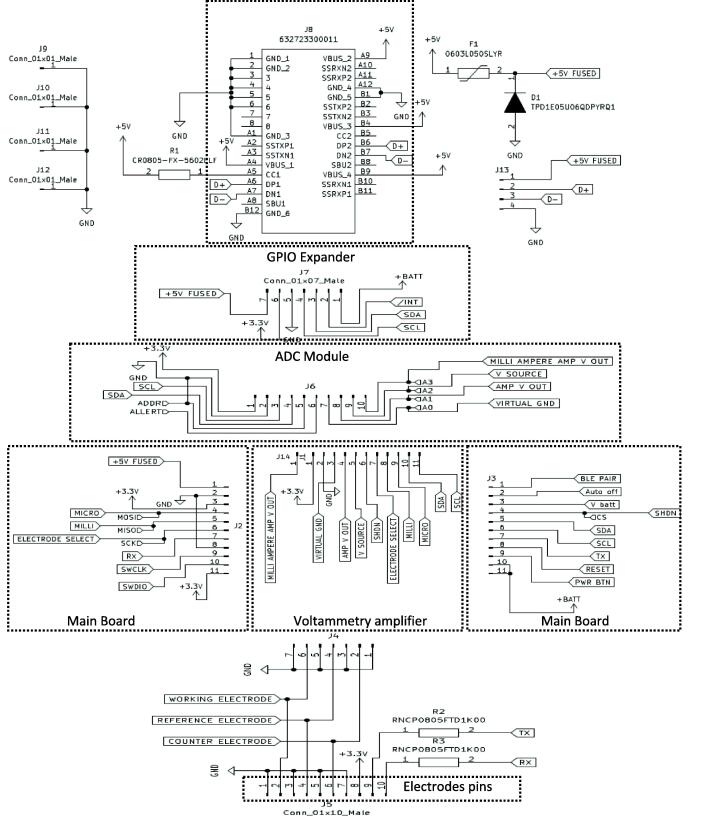
Schematic diagram of link board.

The link board is used to connect all the circuit boards of We-VoltamoStat together. The GPIO expander that attaches to battery charger boards is connected at the back of the link board, while the main board, voltammetry amplifier and ADC module boards are connected at the front. These boards are connected using male-to-female connectors. In addition, a USB connector, 632723300011 (Wurth Elektronik, Germany), has been added to the link board for connection to the battery charger circuit board. The USB support type-C can be used for data transfer and charging the battery, as shown in Fig. 10. This board also supports ten-pin connections for electrodes. Three electrode configurations are formed between pins 1 and 2 for the working electrode, pins 3 and 4 for the reference electrode, and pins 5 and 6 for the counter electrode. In the meantime, pins 7, 8, 9, and 10 are serial port connections for external devices such as a controlling micropump application.

## Design files

See Table 1, Table 2.

**Table 1:** Design Files Summary.

Design file name	File type	Open-source license	Location of the file
Battery Charger.kicad_pcb	PCB layout	CC BY 4.0	http://dx.https://doi.org/10.17632/266vrryspz.3
Battery Charger.kicad_sch	*Schematics*	CC BY 4.0	http://dx.https://doi.org/10.17632/266vrryspz.3
GPIO expander.kicad_pcb	PCB layout	CC BY 4.0	http://dx.https://doi.org/10.17632/266vrryspz.3
GPIO expander.kicad_sch	*Schematics*	CC BY 4.0	http://dx.https://doi.org/10.17632/266vrryspz.3
Voltammetry amplifier.kicad_pcb	PCB layout	CC BY 4.0	http://dx.https://doi.org/10.17632/266vrryspz.3
Voltammetry amplifier.kicad_sch	*Schematics*	CC BY 4.0	http://dx.https://doi.org/10.17632/266vrryspz.3
Main board.kicad_pcb	PCB layout	CC BY 4.0	http://dx.https://doi.org/10.17632/266vrryspz.3
Main board.kicad_sch	*Schematics*	CC BY 4.0	http://dx.https://doi.org/10.17632/266vrryspz.3
Link board.kicad_pcb	PCB layout	CC BY 4.0	http://dx.https://doi.org/10.17632/266vrryspz.3
Link board.kicad_sch	*Schematics*	CC BY 4.0	http://dx.https://doi.org/10.17632/266vrryspz.3
ADS1115.ino	Coding	CC BY 4.0	http://dx.https://doi.org/10.17632/266vrryspz.3
BleSerial.ino	Coding	CC BY 4.0	http://dx.https://doi.org/10.17632/266vrryspz.3
Buttons.ino	Coding	CC BY 4.0	http://dx.https://doi.org/10.17632/266vrryspz.3
Main board.ino	Coding	CC BY 4.0	http://dx.https://doi.org/10.17632/266vrryspz.3
Voltammetry_ampflier.ino	Coding	CC BY 4.0	http://dx.https://doi.org/10.17632/266vrryspz.3
Oled_display.ino	Coding	CC BY 4.0	http://dx.https://doi.org/10.17632/266vrryspz.3
We-VoltamoStat Coding.ino	Coding	CC BY 4.0	http://dx.https://doi.org/10.17632/266vrryspz.3
Casing for We-VoltamoStat.stl	CAD	CC BY 4.0	http://dx.https://doi.org/10.17632/266vrryspz.3
Electrode connector.stl	CAD	CC BY 4.0	http://dx.https://doi.org/10.17632/266vrryspz.3
Bill of Material.xlsx	BOM	CC BY 4.0	http://dx.https://doi.org/10.17632/266vrryspz.3
Result Amplifier Test Accuracy	Raw Data	CC BY 4.0	http://dx.https://doi.org/10.17632/266vrryspz.3
Kodular_App	Android App	CC BY 4.0	http://dx.https://doi.org/10.17632/266vrryspz.3
Operation Video	Mp4	CC BY 4.0	http://dx.https://doi.org/10.17632/266vrryspz.3

**Table 2:** File descriptions.

Design file name	Descriptions
Battery Charger.kicad_pcb	PCB file for the battery charger. This file can be viewed in KiCad software. Some library components are not provided by Kicad but can be downloaded from the websites of EasyEDA and Mouser Electronic. The “Symbol Field Table” in the schematic diagram file can be used to obtain the value of the data components. Then, using the preferences menu button, these downloaded external libraries must be imported into the symbol, footprint, and CAD libraries. In KiCad board design, it is possible to view all board layers (e.g., front, rear, inner, and assembly layers) as well as the 3D viewer design of populated and unpopulated board components. These PCB files are able to be viewed and edited.
Battery Charger.kicad_sch	Schematic diagram file for the battery charger. This file can be viewed in KiCad software. This file has also been packed with data on the used components, such as a datasheet, values, PCB footprint, and a link to retrieve the order with price, manufacturer, and item part number. By designing a circuit diagram, it is possible to define the materials required to construct a bill of materials, while its price depends on the supplier company's price, which can be found on its website.
GPIO expander.kicad_pcb	PCB file for the GPIO expander
GPIO expander.kicad_sch	Schematic diagram file for the GPIO expander
Voltammetry amplifier.kicad_pcb	PCB file for the voltammetry amplifier
Voltammetry amplifier.kicad_sch	Schematic diagram file for the voltammetry amplifier
Main board.kicad_pcb	PCB file for the main board
Main board.kicad_sch	Schematic diagram file for the main board
Link board.kicad_pcb	PCB file for power the link board
Link board.kicad_sch	Schematic diagram file for the link board
ADS1115.ino	Coding for testing the performance of an ADC module when connected to its integrated circuit on a breadboard.
BleSerial.ino	Coding for testing a smartphone's interface through Bluetooth to connect with another device
Buttons.ino	Coding for testing the switch button before modifying it to have a function for Bluetooth or power supply switch on/off.
Power_management.ino	Coding for testing in controlling the ADC of a voltage battery
Voltammetry_ampflier.ino	Coding for testing integrated circuits of analogue signal, transimpedance amplifier, control and buffer amplifier, and noise filtering circuit.
Oled_display.ino	Coding for testing OLED display of battery bar, status Bluetooth connection, QR code and reading of voltage and current
We-VoltamoStat Coding.ino	Combination test program for the ADS1115, BleSerial, buttons, power management and voltammetry amplifier.
Casing for We-VoltamoStat.stl	A 3D CAD enclosure in the.stl format
Electrode connector.stl	A 3D CAD electrode connector in the. stl format
Bill of Material.xlsx	Bill of material for We-VoltamoStat
Result Amplifier Test Accuracy	Raw data of amplifier test-resistor for validation voltage & current accuracy
Kodular_App	Developed Android App in link of c.kodular.io
Operation Video	Step-by-step instructions for using a potentiostat, including how to connect the device to a smartphone, turn it on, pair it, choose a mode, adjust its parameters, take readings, view the results on a graph, store the data, and finally export the raw data to an Excel file for further analysis.

## Bill of materials

The bill of materials (BOM) in this work is divided into six parts of the electronic and 3D printed enclosure. The hardware parts include all the required components, such as SMC electronic components, LiPo charger battery and its casing, printed-circuit boards, ADC module, and pin connectors. All electronic components listed on the BOM are commonly available from standard suppliers and can be easily ordered with the provided data under the filename “Bill of Material.xlsx”. The total cost of the system is 114.44 USD, which is far less than a few open-source and commercial systems. PCB development, including fabrication and component placement fees, is not included in the overall price. It is optional whether to hand-solder or use available services. However, the cost of this service is still cheap, depending on factors like the number of components and the complexity of the circuit's diagram. Meanwhile, the 3D-printed enclosure costs only a few dollars, depending on service charges.

## Build instructions

**Fig. 11:**
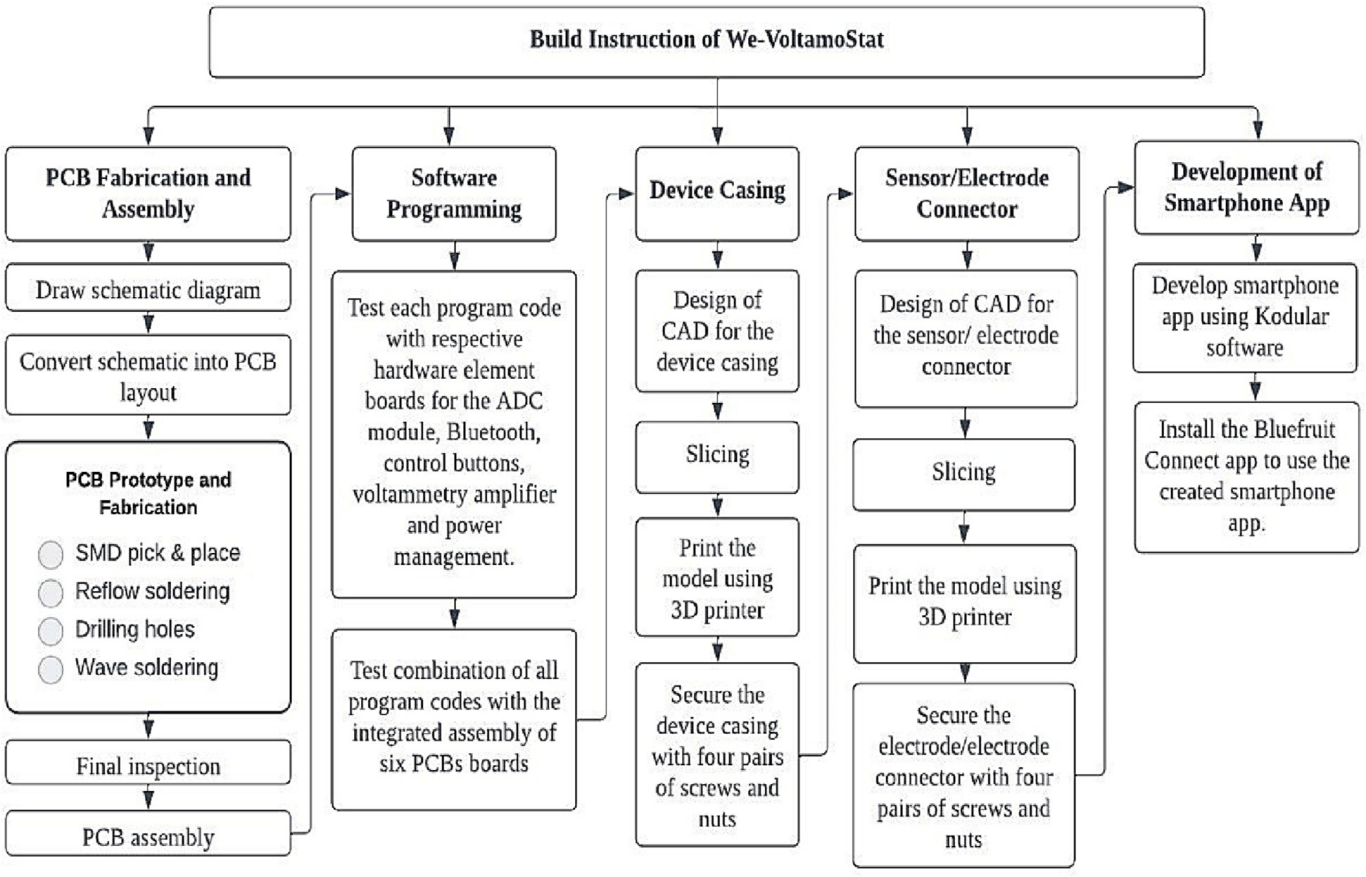
Instruction process of building We-VoltamoStat.

The build instructions section involves PCB fabrication and assembly, software programming, device casing, sensor/electrode connector, and development of smartphone app. Fig. 11 shows the flowchart of the summary of the process of building instructions of We-VoltamoStat.

• PCB fabrication and layout

**Fig. 12:**
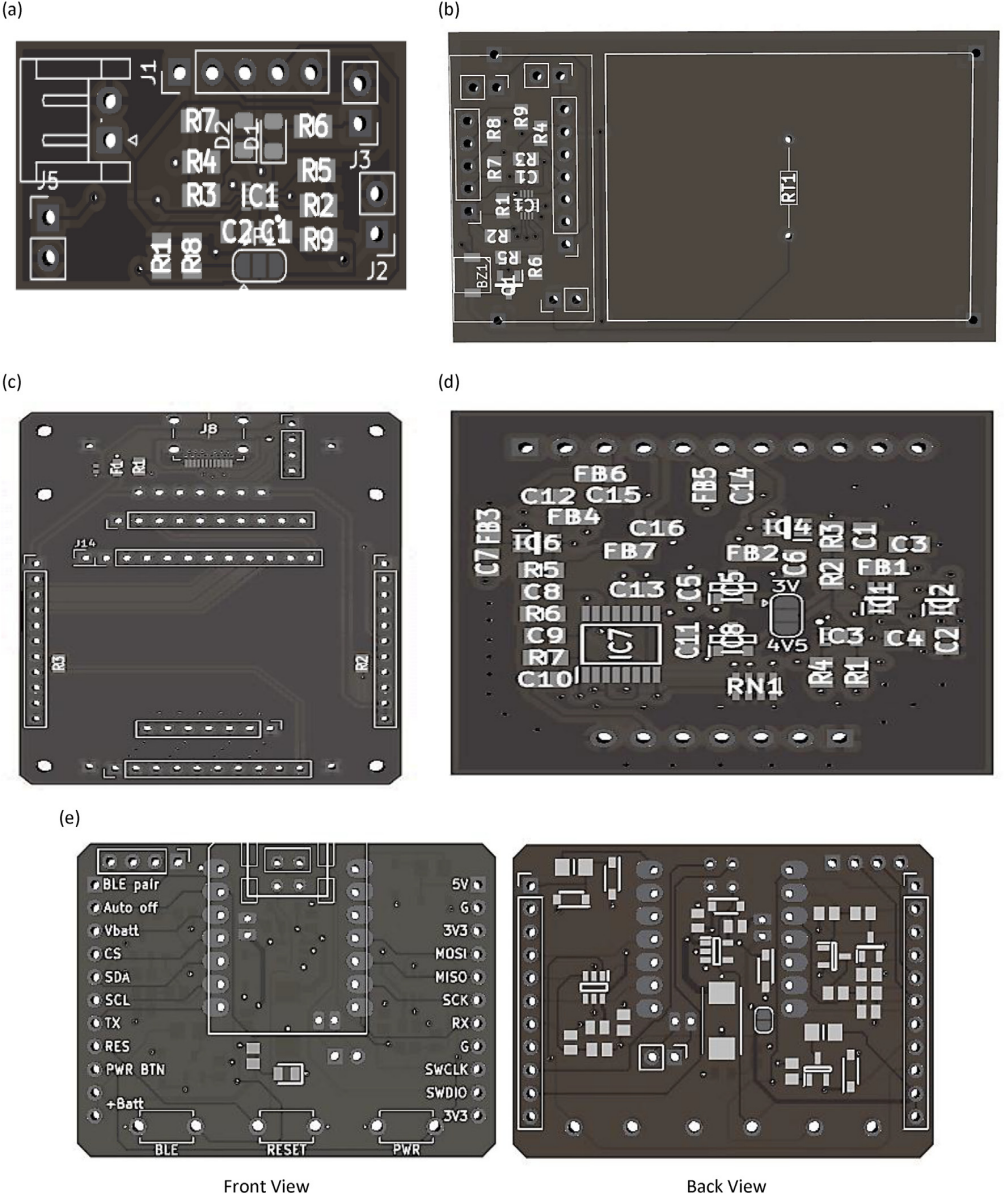
3D layout viewer of unpopulated components of PCB for (a) battery charger, (b) GPIO expander, (c) link board, (d) voltammetry amplifier and (e) main board with font and back view.

**Fig. 13:**
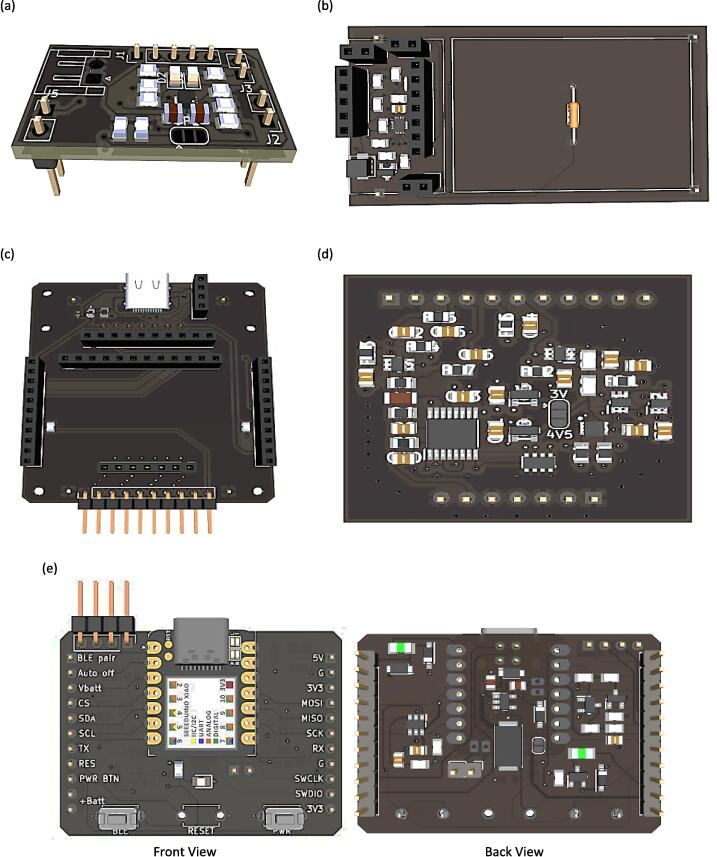
3D layout viewer of populated components of PCB for (a) battery charger, (b) GPIO expander, (c) link board, (d) voltammetry amplifier and (e) main board with font and back view.

**Fig. 14:**
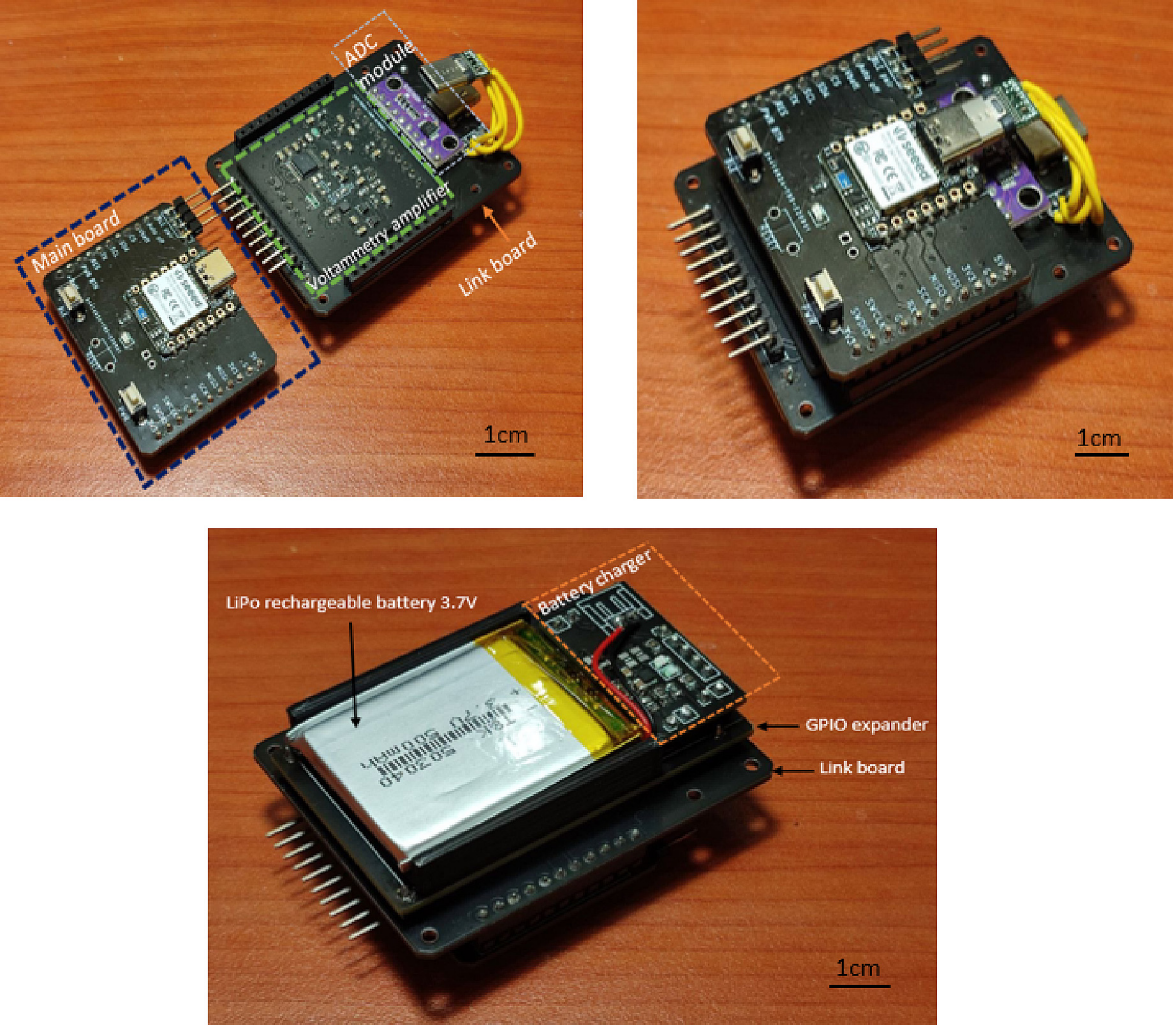
Assembly of four separate pieces of hardware integrated onto a link board.

The Surface Mount Technique (SMT) is used to install components on the Printed Circuit Boards (PCBs). This method utilizes Surface Mount Components (SMCs), which are smaller in size than conventional components, cost-effective, durable, and boost the overall density of the board. Fig. 12 shows the footprint pads for SMCs on unpopulated boards. The design files for the PCB of each peripheral element circuit board can be found in the design files (filenames are in the “.kicad_pcb” format). KiCad software, which is free to use, is employed to create circuit diagrams, including the components layout on single and two-layer PCBs. As shown in Fig. 13, SMT allows SMCs to be placed on both sides of a PCB for the main board, while the rest of the circuits are placed on a single side. All PCB designs have been fabricated using PCB fabrication and assembly services. Fig. 14 shows the actual fabricated PCBs with installed components on the board. The yellow USB wire on the link board has been added to provide a power connection to the Seeeduino Xiao port. In addition, a commercial ADC module has also been introduced to the link board. Battery chargers have been combined with a GPIO expander at the top through male–female pin connectors. A red wire connects an external LiPo charger to the battery charger board's input pin. The internal and external batteries have been charged by attaching them to a USB port of the power source.

• Software Programming

In this work, the program is written using C/C++ programming in the Arduino Integrated Design Environment (IDE), which is a free and open-source platform. Additionally, it is possible to program online using the Arduino Web Editor or offline using the IDE. The program codes for single or combined command functions of the ADC module, Bluetooth, control buttons, voltammetry amplifier, and main board can be found in the design files (filenames are in the “.ino” format). All program codes and the performance of the integrated boards have been tested for functionality.

• Device Casing

**Fig. 15:**
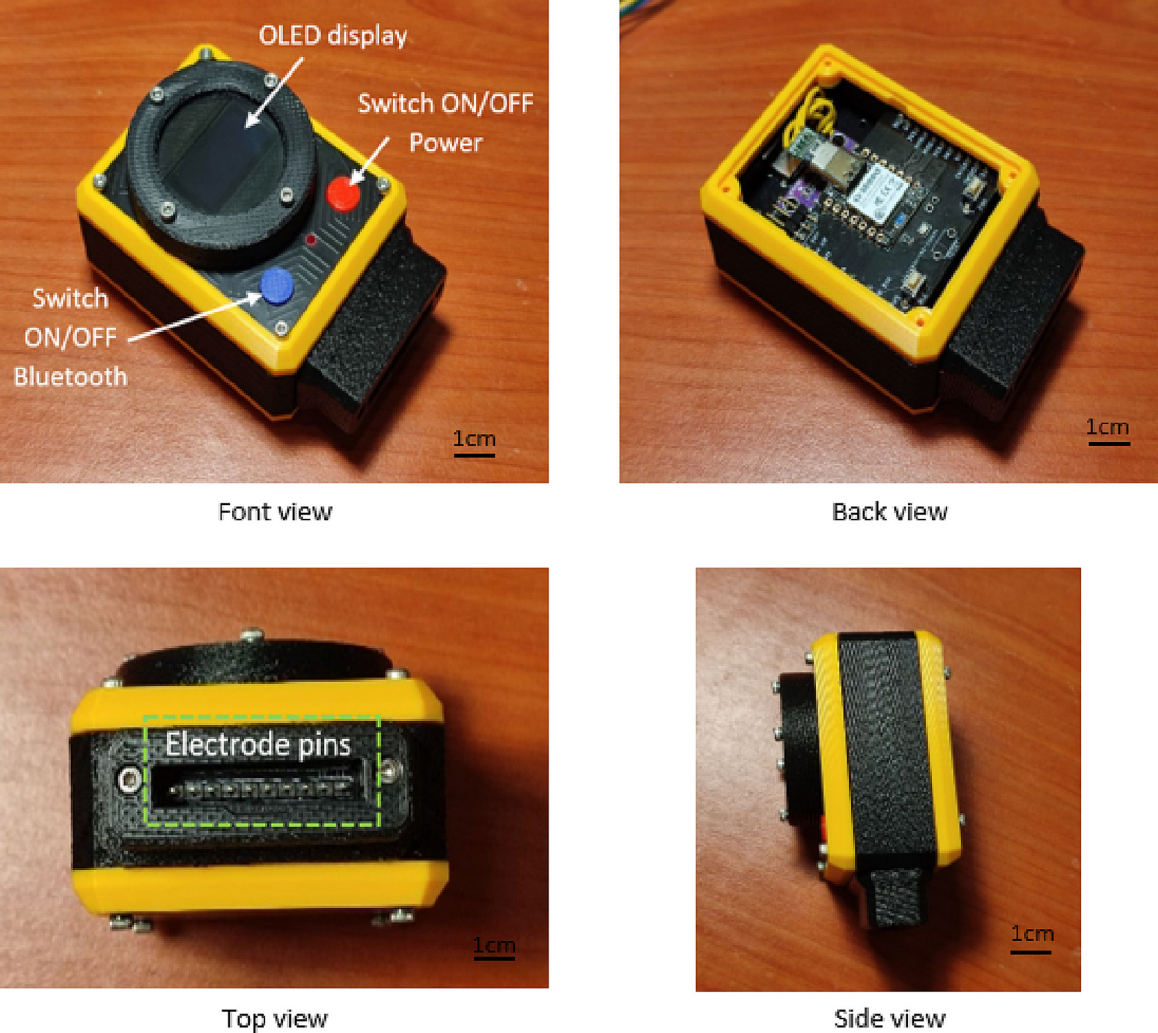
3D printed casing.

DesignSpark Mechanical software was used to design the We-VoltamoStat's device casing and then exported as an STL file. The CAD software has many advanced features, and most importantly, it is free to use. The Creality Slicer software was used to convert the STL file into a specific format file and to establish detailed instructions for the 3D printer to print the object. The casing is made from acrylonitrile butadiene styrene (ABS) and printed with a low-cost Creality Ender-6 3D printer. The casing is lightweight, comfortable, and durable enough for a rough environment, making it suitable for wearable applications. Each of the four pairs of screws and nuts are attached to the casing lid of the entire cover and OLED screen holder to secure the assembly boards, as shown in Fig. 15. This CAD design can be found in the filename “3D CAD device casing”.

• Electrode Connector

**Fig. 16:**
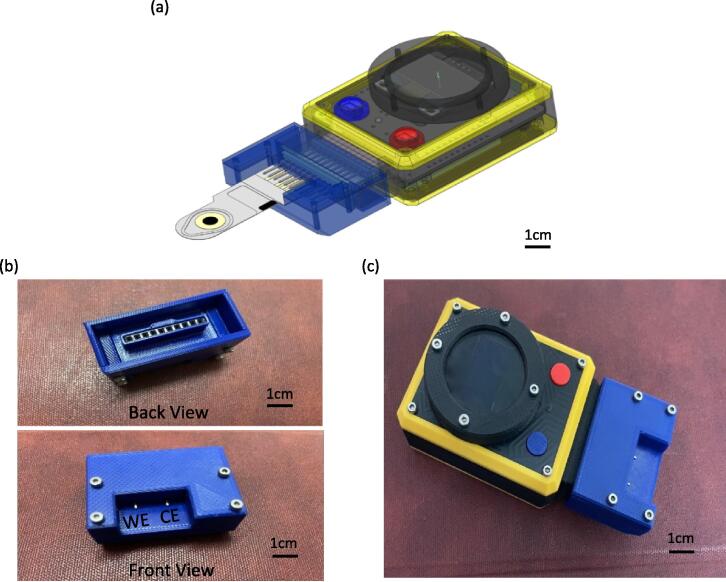
We-VoltamoStat electrode connector (a) CAD model of We-VoltamoStat and example of custom developed sensor, (b) actual printed socket connector and (c) actual developed We-VoltamoStat with sensor socket connector.

The sensor/electrode connector is a socket connector that allows sensors to be connected to the We-VoltamoStat. Fig. 16 shows a 2-electrode configuration of the sensor/electrode socket connector. It was developed using 3D printing, with four pairs of screws and nuts to secure the lid for an electrode holder. It also attaches two electrode pins, WE and CE, for measuring current, while the variable voltage can be set before measurement in the developed smartphone app. The sensor/electrode connector is made from ABS material, which is robust, tough, and safe from electric shock. It was also designed using DesignSpark Mechanical software, which can be found in the filename “3D CAD electrode connector”. Additionally, the socket connector can be designed in various shapes, such as two connectors for a 2-electrode configuration or a 3-electrode configuration sensor. This demonstrates that the We-VoltamoStat is also upgradeable in terms of its socket connector to different designs, depending on its electrode configuration, size, model of commercial electrode sensor and functions.

• Development of Smartphone App

The We-VoltamoStat can be connected to any Android smartphone using Bluefruit Connect (Adafruit, USA). It is available for free download from the Google Play store, where the developed app in Kodular software, named “Kodular_App”, can be installed. Kodular allows for the creation of Android apps without coding, using a quick and simple process of dragging and dropping. Therefore, it is chosen as the method for making open-source design more practical. The smartphone app for the We-VoltamoStat has two screen pages: the 'Bluetooth device' page for establishing a connection and the 'Voltammetry methods' page for configuring input, analyzing raw data from the potentiostat, displaying results graphically, saving data, and uploading it to the cloud. In Kodular, two measurement modes have been characterized: Linear Sweep Voltammetry (LSV) and Cyclic Voltammetry (CV). Each mode consists of the user's experiment parameters being applied to custom data points in standard curve analysis.

## Operation instructions

**Fig. 17:**
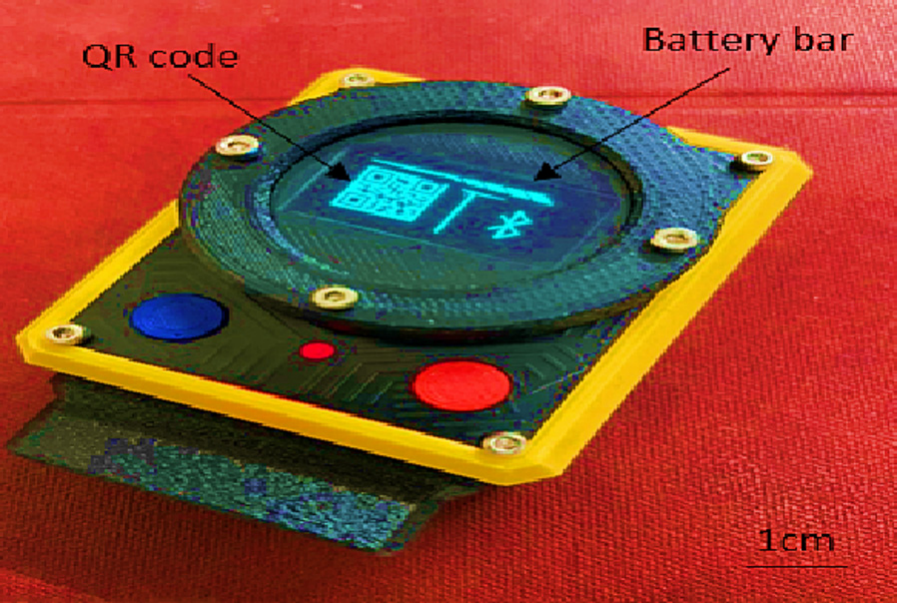
We-VoltamoStat's OLED displays.

**Fig. 18:**
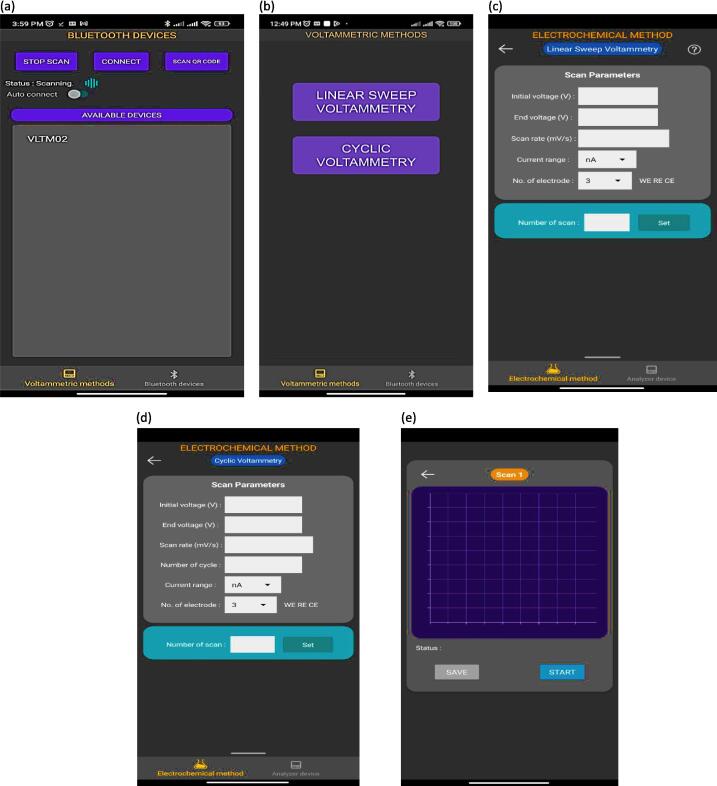
Smartphone screen display designed features in ‘Bluefruit Connect’ app for (a) Bluetooth part of pairing with a potentiostat, (b) selection icon of mode measurements, (c) detail of specifications inputs for selection LSV mode (d) detail of specifications inputs for selection CV mode, and (e) graphical output and save data.

The system has two modes of operation: measurement and analysis. In measurement mode, the potentiostat measures the voltage and current of an electrochemical sensor. The data is then transferred through Bluetooth to a connected smartphone app, where users can visualize or store it. The smartphone and device are paired using a QR code displayed on the device's OLED display or by the device name “VLTM02,” as shown in Fig. 17 and Fig. 18(a) respectively. After successful pairing, the smartphone screen displays “Voltammetry methods” and the user can select the measurement technique: “Linear Sweep Voltammetry” or “Cyclic Voltammetry” as shown in Fig. 18(b). Before starting the measurement process, the user must specify the measurement parameter values. For example, the “Linear Sweep Voltammetry” icon parameters include the start voltage, stop voltage, number of steps, step delay, current range, and number of electrodes, as shown in Fig. 18(c). The potentiostat device will also be configured based on the parameters set in the app. The potentiostat then transmits all electrochemical data output measurements to the “Bluefruit Connect” app for post-processing. The data is displayed graphically on the smartphone screen, while its raw data is simultaneously displayed on the device's OLED display. The measurement data can also be shared and stored in cloud drives (such as via email, Google Drive, or social media) for backup or remote post-processing. The OLED display shows a continuous reading of the sensor, as well as the battery life and Bluetooth connection status (see Fig. 17).

## Validation and characterization

• Voltammetry circuit test

To determine the reliability of the results provided by the potentiostat, it is essential to conduct an accuracy test of the developed circuit. Specifically, the performance of the potentiostat under the CV and LSV modes should be evaluated in terms of parameters such as the voltage source and measured current. This evaluation will help to ensure the accuracy and precision of the potentiostat's performance. Here are the methods and system operations for validating the performance of the voltage source and current in the We-VoltamoStat:

Voltage Source Setting

i. Use the smartphone app to set the voltage source of the device between −1500 to 1500 mV with an increment of 100 mV, and send this information to the device.
ii. The voltage source measured in the potentiostat is known as the output voltage.
iii. The ADC module measures the output voltage and varying voltage source within the range and step increment.
iv. The voltage readings for set voltage and output voltage are displayed in the smartphone app.
v. Store the result into a cloud drive.

Current Setting

i. Connect the WE and CE pins of the We-VoltamoStat to male connectors of a load resistor.
ii. Repeat the procedure in the voltage source part from i to iv.
iii. The reading of measured currents in the transimpedance amplifier is also displayed in the smartphone app.
iv. Store the result into a cloud drive.
v. Measure the calculated current using Ohm’s law.

The accuracy of the voltammetry transimpedance amplifier can be evaluated using the output voltage source. To calculate the current, the output voltage source is divided by the test-resistor value of the object or sample. Mercer et al. [26] and Meloni et al. [29] have implemented a validation method using a test-resistor, or known as the Ohm's law test, to measure current when varying the voltage source range. The voltammetry's measured current is the programmable current measured on the voltammetry amplifier board. The voltage source for the set voltage voltammetry ranges from −1500 mV to 1500 mV with a resolution of 100 mV.

The performance of the transimpedance amplifier can be evaluated using external test-resistors with varying resistance values (999 Ω, 50 Ω, and 999 kΩ) to determine its ability to detect currents in the nano-, micro-, and milli-ampere ranges. The small current unit is specifically designed for highly sensitive detection of analyte chemicals present in low concentrations. In addition to evaluating the current detection capabilities of the transimpedance amplifier, this test can also be useful for calibrating sensors in the future.

**Fig. 19:**
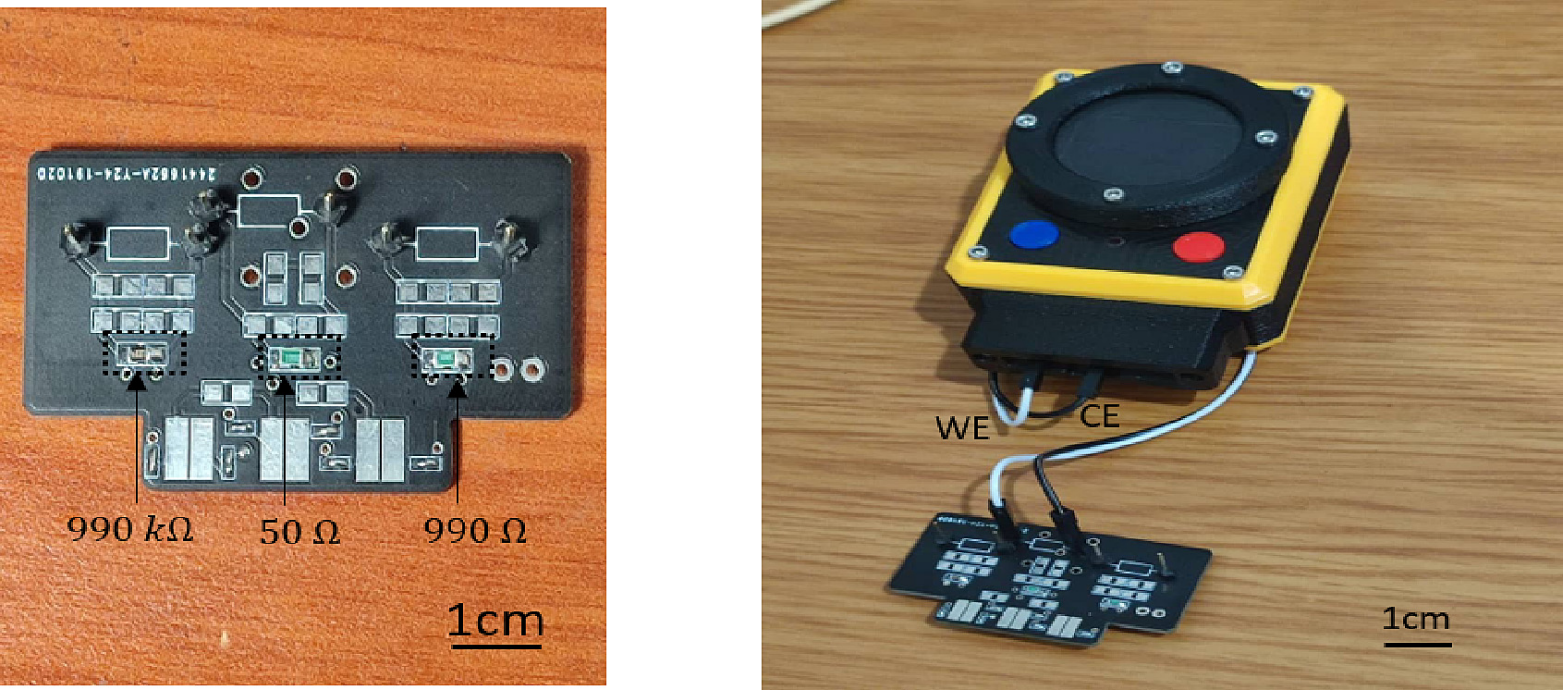
The connection We-VoltamoStat with the SMD resistor board.

Fig. 19 shows the connection setup of the We-VoltamoStat with the SMD test-resistor board. A 999 kΩ test-resistor is set to demonstrates current readings in nano-ampere. This result demonstrates the potentiostat’s ability to measure extremely low currents, making it suitable for use in highly resistive liquids or samples with low target analyte concentrations. A 999 Ω test-resistor is set to measure current in the micro-ampere range, and a 50 Ω test-resistor is set to measure current in the milli-ampere range. The raw data of calculated current and the results of measured currents can be found in the design file, under the file name “Result Amplifier Test Accuracy”.

**Fig. 20:**
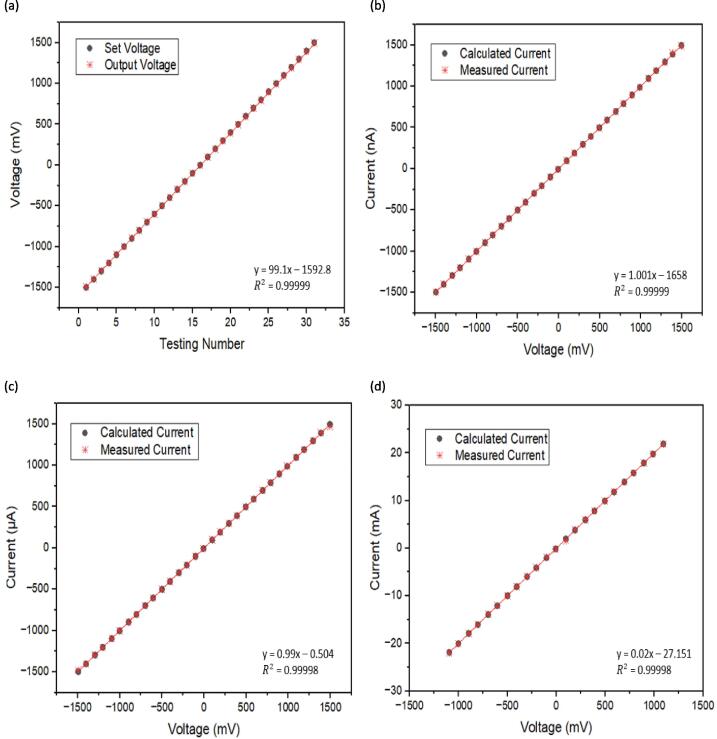
Accuracy test result (a) voltage source, (b) nano-ampere test, (c) micro-ampere test, and (d) milli-ampere test.

Fig. 20 (a) shows the voltage source readings have a strong correlation between the set and output voltage and a good linear regression model of 0.99. Fig. 20 (b), (c), and (d) show that there is a strong correlation between the measured and calculated values for the test-resistors. This indicates the device has a highly sensitive and accurate measurement with R^2^ of approximately 0.99 for the current range of milli- to nano-Ampere. The result is comparable with Irving et al [6], which demonstrated a high correlation with R^2^ is 0.99 for a linear fit of 20 µA, 200 µA, 20 mA, and 200 mA.  They also using Ohm’s law test by comparing measured values and an accuracy test of voltage and current between their device, MyStat, and a digital multimeter.

**Fig. 21:**
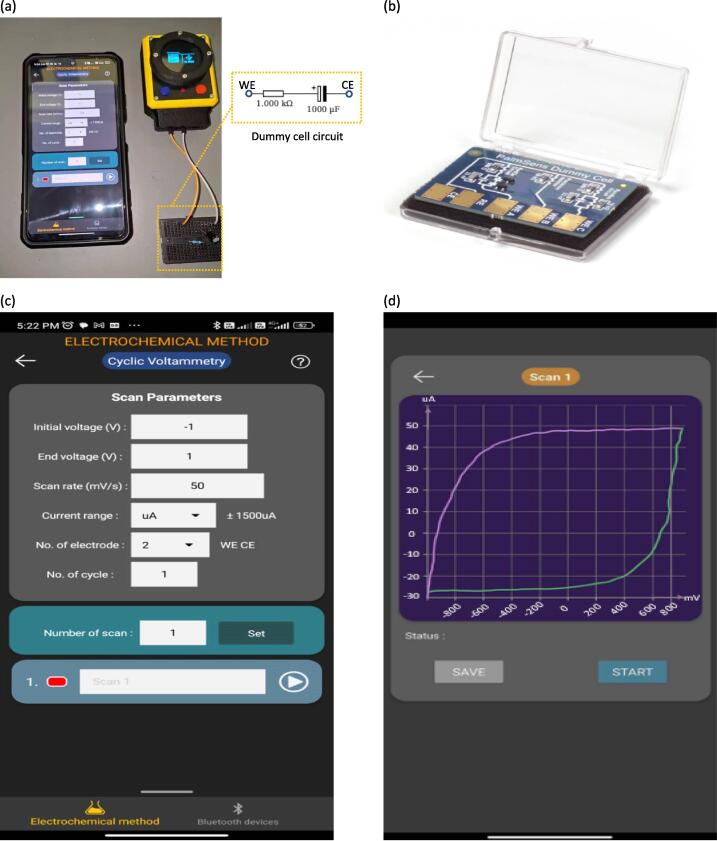
Dummy Cell Testing. (a) Device set-up with connection of potentiostat and dummy cell. (b) Palmsens dummy cell. (c) Parameter values data in CV mode tab view. (d) CV measurement test using dummy cell.

Dummy cells have emerged as a valuable resource for researchers and engineers, offering a simple and effective way to test and calibrate their potentiostats without the need for actual electrochemical cells and eliminating the need for sensors. A dummy cell is typically a straightforward series circuit consisting of a resistor and a capacitor [30], as shown in Fig. 21 (a). These components are connected to the working electrode (WE) and counter electrode (CE) terminals of the potentiostat respectively, replicating the behavior of an actual electrochemical cell. Although the structure might appear simple, it effectively mimics the impedance and response of real cells, allowing for accurate testing and calibration. Moreover, it offer stable and consistent electrical properties, ensuring repeatable and reliable testing results. This allows researchers to identify and address any issues with their potentiostats more effectively. A PalmSens commercial dummy cell can also be used for future calibration, offering a more refined and accurate representation, as shown in Fig. 21 (b). In this work, a cyclic voltammetry (CV) measurement was performed on a dummy cell consisting of a series circuit of a 1 kΩ precision resistor and a 1000 µF capacitor, with scan parameters values as shown in Fig. 21 (c). Fig. 21 (d) shows the resulting current–potential curve exhibiting a trapezoidal shape, similar to the graph result pattern reported by Dobbelaere et al. [7] and Setiyono et al. [31]. This characteristic shape is a result of the exponential decay of current spikes in the dummy cell, which is a characteristic of resistor–capacitor (RC) circuits.

• Battery Lifetime

The We-VoltamoStat's 500 mAh, 3.7 V LiPo battery delivers 15 mA during operating mode and 5 mA during standby mode. The battery's efficiency is considered 100% in ideal conditions. Therefore, it allows for operation without charging for up to 33 h 20 min in operating mode and 100 h in standby mode before the battery is completely drained. The calculations as below:

(1) $$\mathrm{Battery}\;life\;\left(operating-mode\right)=\frac{\mathrm{Battery}\;capacity}{\mathrm{Curent}}=\frac{500\;mAh}{15\;mA}=33\;hrs\;20\;min$$

(2) $$\mathrm{Battery}\;life\;\left(standby-mode\right)=\frac{\mathrm{Battery}\;capacity}{\mathrm{Curent}}=\frac{500\;mAh}{5\;mA}=100\;hrs$$

## Conclusion

The We-VoltamoStat has been designed to make it suitable for portable and wearable real-time electrochemical diagnostics capabilities. The developed is small in size, lightweight, rechargeable battery, tough design, low power consumption, wireless operation, and reliable for voltammetry measurements. On the other hand, it allows the user to easily upgrade the functionality, such as adding more sensing channels, making it possible to simultaneously conduct more than one voltammetric experiment in a single device. Its design and development involved high-quality, cost-effective components and, most importantly, the use of open-source and free software such as the Arduino IDE, Kodular, and the Bluefruit Connect app. The We-VoltamoStat was found to provide high sensitivity and accurate measurements of key parameters such as voltage and current, with a sensitive measurement unit that ensures accuracy in CV and LSV operations. n the future, the device will feature both Bluetooth and Wi-Fi capabilities. Using Wi-Fi, users can link up devices anywhere with larger bandwidth, making it more flexible. The developed app should be compatible with various operating systems, including Android and iOS. A potentiostats should also be able to measure various techniques, such as chronoamperometry and electrochemical impedance spectroscopy, as well as multiple sensor measurements. Thus, it is suggested to include these techniques to provide wide range of measurement. By incorporating machine learning algorithms, data analysis and interpretation can be improved. The open-source design of We-VoltamoStat allows users, researchers, and other interested parties to use and enhance the device's functionality and applications.

## Declaration of Competing Interest

The authors declare that they have no known competing financial interests or personal relationships that could have appeared to influence the work reported in this paper.
